# Therapeutic efficacy of dihydroartemisinin–piperaquine for the treatment of uncomplicated *Plasmodium vivax* malaria in Seacha area, Arbaminch Zuria District, South West Ethiopia

**DOI:** 10.1186/s12936-022-04380-7

**Published:** 2022-11-27

**Authors:** Hussein Mohammed, Heven Sime, Henok Hailgiorgis, Kale Gubae, Mebrahtom Haile, Hiwot Solomon, Kebede Etana, Samuel Girma, Worku Bekele, Melkie Chernet, Getachew Tollera, Geremew Tasew, Bokretsion Gidey, Robert J. Commons, Ashenafi Assefa

**Affiliations:** 1 Bacterial, Parasitic and Zoonotic Diseases, Ethiopian Public Health Institute, Addis Ababa, Ethiopia; 2 Department of Pharmacy, College of Health Sciences, Debre Markos University, Debre Markos, Ethiopia; 3 National Malaria Elimination Programme, Ministry of Health, Addis Ababa, Ethiopia; 4 USAID, Addis Ababa, Ethiopia; 5 World Health Organization, Addis Ababa, Ethiopia; 6 Global Health Division, Menzies School of Health Research, Charles Darwin University, Darwin, Australia; 7 General and Subspecialty Medicine, Grampians Health, Ballarat, Australia; 8 Institute for Global Health and Infectious Diseases, University of North Carolina at Chapel Hill, Chapel Hill, NC USA

**Keywords:** Chloroquine, Dihydro-artemisinin–piperaquine, Efficacy, Ethiopia, *P. vivax*

## Abstract

**Background:**

Declining efficacy of chloroquine against *Plasmodium vivax* malaria has been documented in Ethiopia. Thus, there is a need to assess the efficacy of alternative schizontocidal anti-malarials such as dihydroartemisinin–piperaquine (DHA–PPQ) in *P. vivax* malaria-infected patients. This study was conducted to evaluate the therapeutic efficacy of DHA–PPQ drug in South West Ethiopia.

**Methods:**

This is a single-arm, prospective therapeutic efficacy study in patients with uncomplicated *P. vivax* malaria. The study was conducted from May 2021 to August 2021, based on the standard World Health Organization study protocol for surveillance of anti-malarial therapeutic efficacy. The study endpoint was adequate clinical and parasitological response on day 42.

**Results:**

A total of 86 patients with uncomplicated vivax malaria were enrolled. Of these, 79 patients completed the scheduled follow up; all showing adequate clinical and parasitological responses to day 42, with a successful cure rate of 100% (95% CI 96–100). Parasitaemias were cleared rapidly (86% by day 1 and 100% by day 3), as were clinical symptoms (100% by day 1). Gametocyte carriage decreased from 44% on Day 0 to 1% on day 1 and 0% on Day 2. Mean haemoglobin concentrations increased between day 0 (mean 12.2 g/dL) and day 42 (mean 13.3 g/dL). Treatment was well tolerated and no severe adverse events were observed.

**Conclusion:**

In summary, treatment with DHA–PPQ demonstrated excellent efficacy for uncomplicated *P. vivax*, with no recurrences to day 42, and no safety concerns. This treatment, which is also effective against *P. falciparum*, appears to be an ideal alternative for *P. vivax* as part of the malaria elimination programme.

## Background

In Ethiopia, the scale-up of anti-malarial interventions has been associated with a reduction in malaria cases and deaths in the last decade. Ethiopia’s National Malaria Indicator survey reported a very low prevalence of malaria by microscopy (0.5%) [1], and the country aims to achieve malaria elimination by 2030 [2]. Malaria prevalence varies according to geographical location, with ~ 60% of the population living in areas at risk of malaria.

*Plasmodium vivax* is a widely distributed parasite worldwide [3], but in Africa is essentially restricted to the horn of Africa. Ethiopia is one of the countries co-endemic for *Plasmodium falciparum* and *P. vivax*, with relative proportions of about 70% and 30%, respectively [4]. These proportions vary from place to place and season to season. *P. vivax* develops gametocytes substantially earlier than *P. falciparum*, leading to the early release of gametocytes into the bloodstream from the liver before the appearance of clinical symptoms and facilitating transmission of vivax malaria. Hypnozoite relapse of *P. vivax*, after resolution of the initial infection, further complicates the control and elimination of *P. vivax* compared with *P. falciparum*.

Chloroquine was used for decades as the first-line treatment for all malaria species in Ethiopia. Widespread declines in the efficacy of chloroquine to treat *P. falciparum* led to its replacement with sulfadoxine-pyrimethamine (SP) in 1998 [5, 6]. Since 2004, artemether–lumefantrine (AL) has been used for uncomplicated falciparum malaria, but chloroquine continues to be the first-line blood stage treatment for vivax malaria. The alternative treatment for *P. falciparum* or *P. vivax* is quinine [7]. However, quinine has frequent side effects and the need for a 7-day treatment course.

Unlike *P. falciparum*, *P. vivax* has the ability to form dormant hypnozoites in the liver that can cause a relapse of infection weeks to months after the initial attack [8]. About 80% of recurrent *P. vivax* infections are derived from hypnozoites rather than new infections [9]. Currently, primaquine is used to kill hypnozoites and prevent relapses. It is used in combination with chloroquine, as radical cure treatment. In 2022, Ethiopia adopted the use of directly-observed primaquine for radical cure of *P. vivax* without prior glucose-6-phosphate dehydrogenase (G6PD) testing in regions with all transmission levels [10]. In Ethiopia, dihydroartemisinin–piperaquine (DHA–PPQ) is recommended as a second-line treatment for both *P. falciparum* and *P. vivax*. This drug is approved by the World Health Organization (WHO) and registered in Ethiopia [10].

Several studies have reported a decline in the efficacy of chloroquine against *P. vivax* in Ethiopia [11–14] highlighting the importance of having alternative blood stage options. DHA–PPQ is one such option. This artemisinin-based combination therapy is safe, very well tolerated and has high cure rates [15]. The slow elimination of piperaquine provides a further benefit for *P. vivax*, with prolonged post-treatment prophylaxis beyond that provided by artemether-lumefantrine, suppressing the risk of recurrence for 6–8 weeks after treatment [16, 17].

There are a lack of data on the effectiveness of DHA–PPQ for the treatment of *P. vivax* in Ethiopia. Such data are important to understand the potential utility of DHA–PPQ in Ethiopia as an alternative to chloroquine or artemether-lumefantrine for the treatment of *P. vivax*. This study was conducted to assess the therapeutic efficacy and safety of DHA–PPQ for the treatment of vivax malaria in the Seacha area of Ethiopia.

## Methods

### Study site and design

**Fig. 1:**
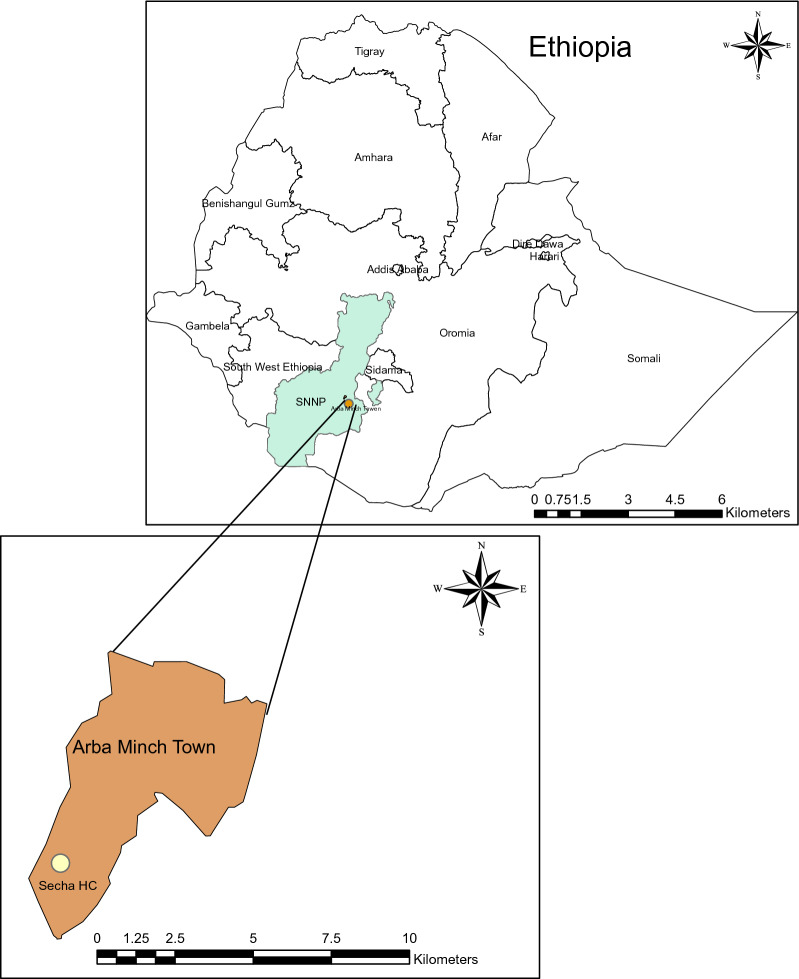
Map of the study site

The study was conducted in Seacha area, Arba Minch Zuria District, located in the Gamo Gofa zone of the Southern Nations, Nationalities, and Peoples’ Region of Ethiopia (Fig. 1). The study area is located about 500 km south of Addis Ababa in the Greater Rift Valley, at latitude 6° 01′ 59.99′′ N and longitude 37° 32′ 60.00′′ E, at an elevation of 1285 m above sea level. The average annual temperature is 29.7 °C and the average annual rainfall is 700 mm. Malaria transmissions in the study area is highly seasonal and markedly unstable with an entomological inoculation rate of 71.1 infectious bites per person per year [18]. *Plasmodium vivax* is the predominant parasite species and *Anopheles arabiensis* is the major vector. Based on the current malaria stratification and mapping of the country [19], this study area is considered an area of moderate (altitude range from 1000 to 1750 m) *P. vivax* transmission.

### Study population, inclusion, and exclusion criteria

Febrile patients visiting the outpatient department of the Seacha Health Centre who fulfilled the inclusion criteria set by the WHO protocol for the assessment of the therapeutic efficacy of anti-malarial drugs were eligible for inclusion [20]. Inclusion criteria were age 6 months or older, fever (axillary temperature ≥ 37.5° C) or a history of fever in the preceding 24 h, microscopically confirmed *P. vivax* mono-infection, with an asexual parasitaemia of 250 parasites/μL or above and living within the facility catchment area (i.e. < 5–10 km radius of the health centre). Participants were excluded if they were pregnant, breast-feeding, had mixed species infection (*P. falciparum* and *P. vivax*), had a day 0 haemoglobin < 5.0 g/dL, were unable to take oral medication or had repeated vomiting, had known hypersensitivity to the study drugs, had evidence of severe malaria, heart or liver diseases, had severe malnutrition, or had evidence of a non-malarial febrile illness (i.e. otitis media, tonsillitis, measles, acute lower respiratory tract infection, severe diarrhoea with dehydration). Primaquine therapy was not provided until the day of first recurrence or day 42.

### Sample size determination

The sample size was determined according to the WHO protocol [20]; using the single population proportion formula and calculated assuming a 5% margin of error, 95% confidence interval (CI). A minimum sample size of 73 was calculated*,* with a 20% increase to allow for loss to follow-up and withdrawal during the 42-day follow-up period, at least 88 patients were to be recruited.

### Treatment and follow-up

Eligible patients were treated with a full three-day course of DHA–PPQ. DHA–PPQ (DHA 40 mg, PPQ 320 mg per tablet, Guillin Pharmaceuticals China; Bach no. SQ200201) was provided by the Ethiopian Federal Ministry of Health (FMoH) through WHO support. Daily drug dosage was based on body weight as follows: half a tablet, 5 to < 8 kg; three-quarters of a tablet, 8 to < 14 kg; one tablet, 14 to < 25 kg; two tablets, 25 to < 36 kg; three tablets, 36 to < 60 kg, four tablets 60 to < 80 kg and five tablets, > 80 kg.

All medication doses were given under the direct supervision of the study team. Medication given to young children was crushed, mixed with water, and administered as a suspension. Patients were observed for thirty minutes to 1 h after each dose to monitor for vomiting or other side effects. If vomiting occurred within 30 min after administration, the full dose was re-administered. If vomiting occurred between 30 min and 1 h after administration, half of the dose was re-administered. Patients who vomited a second time were withdrawn from the study and received parenteral therapy according to national guidelines. Standard dose primaquine (0.25 mg/kg daily for 2 weeks) was given to patients at the end of the follow-up period or at recurrence.

Patients were followed-up daily for the first 3 days after the first dose (day 0) and then weekly on day 7, 14, 21, 28, 35, and 42. Clinical and laboratory evaluations were undertaken during each follow-up visit. Patients were also assessed on an unscheduled visit if symptoms occurred. Adverse events and severe adverse events were defined according to the WHO protocol for monitoring the therapeutic efficacy of anti-malarial drugs and were monitored at each follow‐up visit [20]. Fever clearance and parasite clearance were assessed over the first seven days. Late clinical failure (LCF) and Late parasitological failure (LPF) was defined as the reappearance of parasitaemia between day 4 and day 28 (or 42) with fever and without, respectively, and without previously meeting any of the criteria for ETF or LCF,

### Laboratory procedures

Thick and thin blood smears were prepared on a single slide, for parasite detection and species identification, respectively. Two smears were prepared from all participants at all follow-up visits. The first slide was prepared by staining with 10% Giemsa for 10–15 min for initial screening. The second slide was stained using 3% Giemsa for 30 min and read by two independent laboratory technicians from the health centre; in case of discrepancy between the first and second readings, a third reading was performed. Parasite densities were recorded for all positive slides. The number of asexual parasites was counted per 200 white blood cells (WBC) and parasitaemia was estimated assuming WBC counts of 8000/µl. Gametocytes were counted against 500 WBCs. Before any blood smear was interpreted as negative, two hundred oil-immersion high power fields on the thick film were read [21]. To ensure microscopy quality, all slides were cross-checked by a WHO-accredited microscopist at the Adama Malaria Control and Monitoring Centre. Haemoglobin concentrations were measured using a portable spectrophotometer (HemoCue^®^, Angelholm, Sweden), on days 0, 14, 28, and 42. Female study participants aged 12 years and older were screened for pregnancy on enrolment.

### Statistical analysis

Study endpoints were categorized into primary and secondary endpoints. The primary outcome was the day 42 overall efficacy of DHA–PPQ stated as polymerase chain reaction (PCR)-uncorrected proportion with adequate clinical and parasitological response (ACPR). Secondary endpoints were the incidence of adverse events, fever and parasite clearance, and haemoglobin levels across the follow-up period. Data were double-entered into the WHO Excel spreadsheet designed for therapeutic efficacy data. Data were also entered into IBM SPSS (version 24) software to calculate descriptive statistics (mean, standard deviations, and percentages). Based on WHO guidelines, the adequate clinical and parasitological response on day 42 was calculated using the Kaplan–Meier cumulative risk method [20].

### Ethical approval

The study was approved by the Institutional Review Board (IRB) of the Ethiopian Public Health Institute (EPHI). Written informed consent was obtained from all of the study participants ≥ 18 years of age or by parents or guardians of children less than 18 years of age, with children 12–17 years of age providing written informed assent.

## Results

### Baseline characteristics

**Fig. 2:**
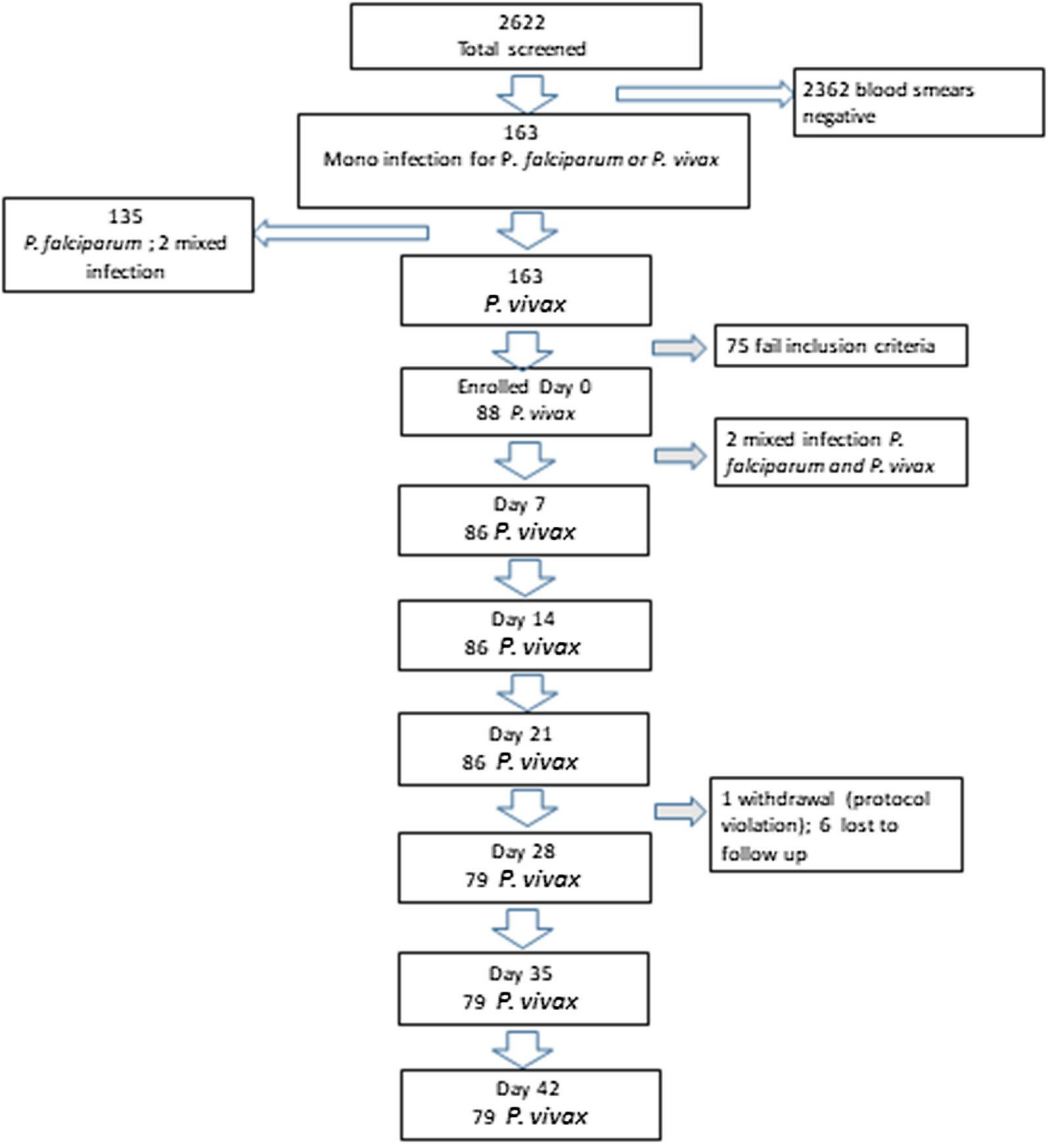
Study profile screening Enrollment and follow-up of patients

A total of 2662 fever cases were screened at a health centre in Seacha site between May 2021 and the end of August 2021 (Fig. 2). Of these 6% were microscopy positive for *P. vivax* (163/2662) of which 88 (54%) were initially enrolled and treated with DHA–PPQ. The main reasons for exclusion from enrolment were living too far away for follow-up (n = 35; 21%), refusal to consent (n = 15; 9%), pregnancy or lactation (n = 10; 6%), concomitant disease (n = 11; 7%) and non-residence (n = 4; 2%). After slide rechecking by the WHO accredited microscopist, 2 further patients were excluded (1 with mixed infection with *P. falciparum*, and 1 with *P. falciparum* mono-infection). In total, 86 patients were finally enrolled and followed-up according to WHO protocols. There were 7 (8%) patients who were censored during follow-up (1 protocol violation and 6 lost to follow-up) (Fig. 2). Table 1 presents the demographic characteristics of the enrolled study patients. 48 (56%) were male and 38 (44%) were female with an age range of 0.7 to 56 years (mean 18.5 [standard deviation 10.9]). The majority of patients were aged ≥ 15 years (n = 53, 62%) (Table 1). The geometric mean parasite density at enrolment was 7477/µL (95% CI 5162–10,480).

**Table 1:** Baseline characteristics of study patients in Seacha Health Centre, South West Ethiopia

Characteristics	Value
Total enrolled	86
Gender, n (%)	
Male	48 (56%)
Female	38 (44%)
Age, years (± SD)	18.5 (10.9)
Age group, n (%)	
< 5 years	7 (8%)
5–14 years	26 (30%)
≥ 15 years	53 (62%)
Body weight, kg (± SD)	43.8 (17.9)
Body temperature, °C (95% CI)	37.9 (37.8–38)
Geometric mean parasitaemia, /µL (95% CI)	7477 (5162–10,480)
Number of patients with gametocytes observed, n (%)	37 (43%)
Haemoglobin concentration, g/dL (± SD)	12.2 (1.4)

n (%); mean (SD); mean (95% CI)

*CI* confidence interval; *SD* Standard deviation; *n* baseline number; *DHA–PPQ* dihydroartemisinin–piperaquine

### DHA–PPQ efficacy

Of the 86 patients enrolled in the study, 79 (92%) of them completed the 42-days follow up. The treatment outcomes by microscopy are presented in Table 2. No recurrent parasitaemia was found within 42 days, with the day 28 and day 42 cure rates 100% (95% CI 96–100).

**Table 2:** The outcomes of DHA–PPQ treatment in patients infected with uncomplicated *P. vivax* malaria

Treatment outcome	DHA–PPQ
Total patients enrolled	86
Total patients treated per protocol (%)	79 (92%)
Early treatment failure, %	0
Late clinical failure, %	0
Late parasitological failure, %	0
Adequate clinical and parasitological response, n (%) [95%CI]	79 (100%) [96–100]

### Gametocyte carriage

The proportion of patients with noticeable gametocytaemia at enrolment was 44% (38/86). The proportion of patients that had no evidence of gametocytes on day-1 was 99% (85/86). No gametocytes were present on day-2.

### Haemoglobin

The mean haemoglobin concentration at baseline was 12.2 g/dL (standard deviation 1.4). There was a small increase in mean haemoglobin concentration from baseline to day 28 and day 42. No study participant developed severe anaemia (< 8 g/dL) during follow up.

### Fever and parasite clearance

**Fig. 3:**
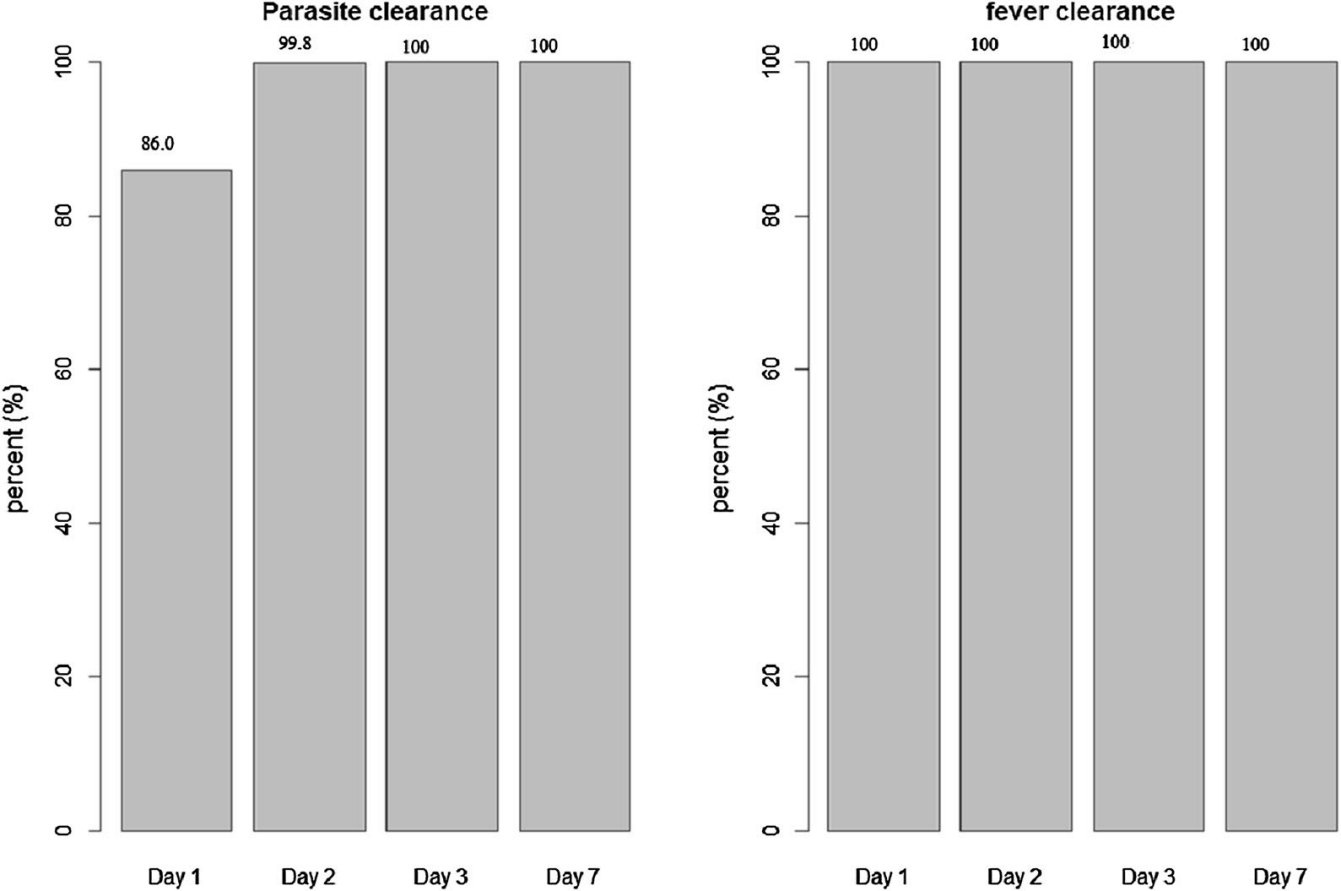
Graphical representation of parasite and fever clearance rate

The mean body temperature at day 0 was 37.9 °C (95% CI 37.7–38.2), with all patients having fever resolution (< 37.5 °C) by day 1 (Fig. 3). Parasites were cleared in 74 of 86 patients (86.0%) at day 1 and 84 of 85 patients (99%) at day 2.

### Adverse events

No severe adverse events were recorded during the study period. DHA–PPQ generally appeared to be safe and well tolerated. 17 adverse events were recorded in 86 patients. The most common adverse events recorded were fever (n = 5, 6%), followed by abdominal pain (n = 3, 4%) and vomiting (n = 3, 4%) (Table 3).

**Table 3:** Adverse events following treatment with DHA–PPQ in Seacha, South West Ethiopia from May–August 2021

Adverse events	DHA–PPQ (n = 86)
Fever	5 (6%)
Vomiting	3 (4%)
Abdominal pain	3 (4%)
Headache	2 (2%)
Skin rash	2 (2%)
Coughing	1 (1%)
Weakness	1 (1%)

## Discussion

This therapeutic efficacy study conducted across all age groups revealed excellent efficacy of DHA–PPQ for the treatment of uncomplicated vivax malaria in a study setting. Parasites were not observed in any patient at 48 h, remaining negative to the end of follow up at day 42, and fever clearance was rapid with all patients afebrile within 24 h. No severe adverse events were reported.

Previous therapeutic efficacy studies of chloroquine have demonstrated high treatment failure rates, with a study in 2015 in southern Ethiopia having failure rates of 3.8% to 21.9% within 28 days and a study in Halaba district in 2013 in southern Ethiopia, having a failure rate of 13% within 28 days [11, 12]. In addition one of the reports cited Yohannes et al. confirmed resistance with demonstration of high CQ blood levels at the time of recurrence [13]. These findings raise the prospect of increasing chloroquine resistance in *P. vivax* throughout Ethiopia, and highlight the need to ensure that alternative treatment options are available. The ongoing monitoring for drug resistance and assessment of alternative options is crucial for treatment guidelines to be revised and for elimination strategies to be planned and implemented.

The present study of DHA–PPQ demonstrated no recurrences prior to day 42. This is consistent with prevention of recrudescence (early recurrence due to failure of blood stage anti-malarial treatment), in addition to prevention of relapses prior to day 42. The absence of recurrent infections prior to day 42 is consistent with a prolonged period of post-treatment prophylaxis following DHA–PPQ [22]. This is secondary to the long terminal elimination half-life of piperaquine, which prevents the emergence of patent parasitaemia for 6–8 weeks [23]. Piperaquine prevents both relapses and reinfections from becoming patent over this period. Co-administration of primaquine with DHA–PPQ will further reduce the risk of relapses after the effect of piperaquine has ceased [24].

Despite a high gametocyte carriage rate detected pre-treatment in the current study (44%), complete gametocyte clearance was observed by day 2. Thus, DHA–PPQ demonstrated good transmission-blocking efficacy, rapidly preventing infection of mosquitoes. In addition, DHA–PPQ can be used to be used to treat both *P. falciparum* and *P. vivax* malaria. In remote malaria endemic areas, where the ability to differentiate *Plasmodium* species may be limited, use of DHA–PPQ may be used to simplify treatment and avoid complications caused by using the wrong anti-malarial.

The day 0 mean haemoglobin level was lower than those on day 28 and day 42. This is consistent with the expected increase in haemoglobin concentration following successful parasite clearance [22]. However, successful blood stage clearance is only partly effective at improving haemoglobin. The prevention of subsequent relapses, through the use of primaquine, can lead to potentially higher haemoglobin concentrations [22].

The generalizability of this study was limited by being undertaken at a single health centre. In addition, it is unclear what the efficacy of chloroquine against *P. vivax* is in the Seacha region and whether the need for an alternative treatment option is urgent.

Effective anti-malarial treatment is a cornerstone of effective malaria control and progression towards achieving elimination in Ethiopia. Therefore, periodic monitoring of currently used anti-malarial drugs and potential alternative ACTs through therapeutic efficacy studies is important to identify the early emergence of anti-malarial drug resistance. The findings from this study demonstrate similar efficacy of DHA–PPQ to a previous unpublished study conducted between 2017 and 2018 in Pawi and Arba Minch (personal communication A Assefa).

## Conclusion

This is the first published study to assess the efficacy of DHA–PPQ against *P. vivax* infection in all ages in Ethiopia. It demonstrates that DHA–PPQ is highly efficacious and safe for the treatment of uncomplicated *P. vivax* malaria in Arba Minch, Ethiopia. With potential increases in chloroquine resistance in vivax malaria in Ethiopia, DHA–PPQ provides an alternative treatment option to quinine, with rapid parasite clearance and resolution of fever and effective gametocyte killing. Further assessment of DHA–PPQ in different transmission settings in Ethiopia will be important to confirm these results.

## Data Availability

The database analysed in this study are available from the corresponding author.

## Abbreviations

ACT: Artemisinin-based combination therapy
ACPR: Adequate clinical and response
AEs: Adverse events
DHA–PPQ: Dihydroartemisinin–piperaquine
CI: Confidence interval
ETF: Early treatment failure
EPHI: Ethiopian Public Health Institute
K-M: Kaplan–Meier
LCR: Late clinical failure
LPF: Late parasitological failure
WHO: World Health Organization
